# The complete chloroplast genome of *Hydrangea davidii* (Hydrangeaceae)

**DOI:** 10.1080/23802359.2020.1828001

**Published:** 2020-10-21

**Authors:** Lie-Fen He, Shao-Juan Qiang, Yong-Hong Zhang, Hong-Fei Li

**Affiliations:** a School of Life Sciences, Yunnan Normal University, Kunming, China; b Beibei Forest Tree Seedling Station, Chongqing, China

**Keywords:** *Hydrangea davidii*, complete chloroplast genome, Hydrangeaceae, phylogenetic analysis

## Abstract

*Hydrangea davidii*, a perennial shrub of Hydrangeaceae, is an ornamental plant endemic to China. Here, we report the complete chloroplast genome of *H. davidii*. The complete chloroplast genome is totally 158,054 bp in length with a typical quadripartite structure. It consists a pair of inverted regions (IRs) of 26,140 bp, which were separated by a large single copy (LSC) region of 87,008 bp and a small single copy (SSC) region of 18,766 bp, respectively. The chloroplast genome encoded 131 genes, including 85 protein-coding genes, 8 rRNA genes and 38 tRNA genes. The GC content in the whole cp genome, LSC region, SSC region, and IR region are 37.8%, 36.0%, 31.7%, and 43.1%, respectively. In total, 49 SSRs were identified in the complete chloroplast genome. Phylogenetic analysis showed that *H. davidii* is closely related to *Hydrangea platyarguta* with a support rate of 100%.

The genus *Hydrangea* Linnaeus (Hydrangeaceae), including ca. 73 species, is mainly distributed in the temperate zone of the northern hemisphere with 33 species (25 endemic) in China (Wei and Bartholomew 2001). *Hydrangea* has been cultivated for gardening plants as well as pot-cultured and cut-flowers worldwide due to its high ornamental value. There are many unique and interesting characters of *Hydrangea* made it famous, such as big inflorescences with both inconspicuous fertile and attractive neuter flowers, flower color change with soil pH degree, long flowering period from June to August (Cerbah et al. 2001; Barbara Conolly et al. 2010; Yue and Peng 2019). *H. davidii* Franchet is a 1–3 m tall perennial shrub distributing in mixed forests on mountain slopes or in valleys of Sichuan, Yunnan and Guizhou of China (Wei and Bartholomew 2001). It has big and dense corymbose cymes with sterile flower borne at margin of inflorescence and has been cultivated for garden plants with high ornamental value. Additionally, it had been reported that *H. davidii*, as one of three origin plants for Traditional Chines Medicine Xiao-Tongcao, had medicinal value on anti-inflammatory and antipyretic and diuretic effects (Shen et al. 1998). In this study, we first report the complete chloroplast (cp) genome of *H. davidii* to provide genomic resource for further research on the phylogenetic reconstruction, genetic diversity and evolution.

The fresh leaves were collected from a healthy *H. davidii* individual, growing in Xiao-Yanfang Natural Reserve, Yongshan County (28°22'15″N, 110°55'22″E) in Yunnan Province. The voucher specimens (XYF-092) was deposited in the Herbarium of Yunnan Normal University. The total genomic DNA was extracted using a modified CTAB protocol (Allen et al. 2006). A pair-end (PE) sequence library was constructed and sequenced using the Illumina HiSeq 2500-PE150 platform (Illumina, USA). Raw sequence data were filtered to obtain clean reads using NGS QC Toolkit version 2.3.3 with default parameters (Patel and Jain 2012). The complete cp genome was de novo assembled by NOVOPlasty (Dierckxsens et al. 2016) and annotated with the online annotation tool GeSeq (Tillich et al. 2017). The SSRs were detected using online software MISA (Thiel et al. 2003). The unit sizes of mono-, di-, tri-, tetra-, penta-, and hexa-nucleotide repeats were set to minimum number of repeats of 10, 5, 4, 3, 3, and 3, respectively.

The complete chloroplast genome of *H. davidii* (GenBank accession number: MT861130) is 158,054 bp in length and has the typical quadripartite structure, including a large single copy (LSC) region of 87,008 bp, a small single copy (SSC) region of 18,766 bp, and two separated inverted region (IRs) of 26,140 bp. The overall GC content of the cp genome is 37.8%, while the corresponding values of the LSC, SSC, and IR regions were 36.0%, 31.7%, and 43.1%, respectively. The cp genome encoded 131 genes, including 85 protein-coding genes (PCGs), 8 rRNA genes and 38 tRNA genes. Among these genes, 61 PCGs and 23 tRNA genes are located in the LSC region (including one interregional gene *rps*19), while 12 PCGs and one tRNA gene occur in the SSC region (including two interregional genes *ycf*1 and *ndh*F). All the eight rRNA genes are duplicated in the IR regions. Each of the IR regions contains six PCGs and seven tRNA genes. A total of 16 genes contains a single intron, and two genes (*ycf*3 and *clp*P) contain two introns. 49 SSRs were identified in the complete chloroplast genome in total. The numbers of mono-, di-, tri-, tetra-, penta-, and hexa-nucleotide nucleotides SSRs are 40, 2, 2, 3, and 2, 0, respectively and mono-nucleotide A/T SSR motif was most frequent.

To analyze the phylogenetic location of *H. davidii*, 21 species of Hydrangeaceae and four outgroups were aligned by the MAFFT version 7 software (Katoh and Standley 2013). Maximum-likelihood (ML) tree was constructed using RAxML 8.2.11 (Stamatakis 2014) with the GTR + G nucleotide substitution model and all branch nodes were calculated under 1,000 bootstrap replicates. The phylogenetic analysis revealed that all sampled species of Hydrangea were clustered into one monophyletic clade with a high bootstrap value. Within *Hydrangea*, *H. davidii* is closely related to *H. platyarguta* with a support rate of 100% (Figure 1).

**Figure 1.**
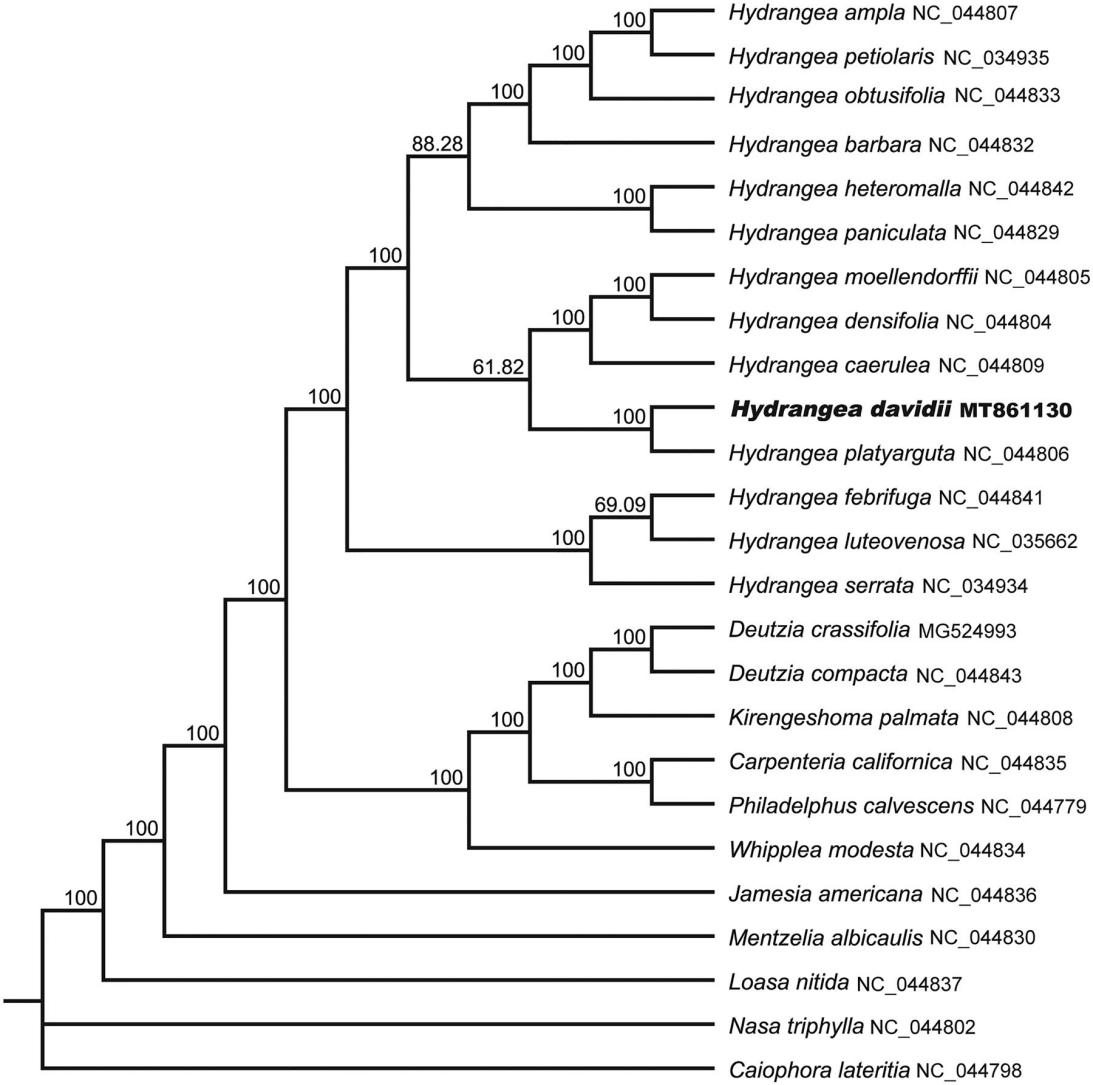
Maximum-likelihood (ML) tree of *H. davidii* and its congeners based on the complete chloroplast genome sequences. Bootstrap values from 1000 replicates were shown next to the nodes.

## Data Availability

The data that support the findings of this study are openly available in NCBI at https://www.ncbi.nlm.nih.gov/, reference number [MT861130] [SRR12526392], or available from the corresponding author.
